# Molecular evolution and functional divergence of the bestrophin protein family

**DOI:** 10.1186/1471-2148-8-72

**Published:** 2008-02-28

**Authors:** Vladimir M Milenkovic, Thomas Langmann, Rainer Schreiber, Karl Kunzelmann, Bernhard HF Weber

**Affiliations:** 1 Institute of Human Genetics, University of Regensburg, Regensburg, Germany; 2 Institute of Physiology, University of Regensburg, Regensburg, Germany

## Abstract

**Background:**

Mutations in human bestrophin 1 are associated with at least three autosomal-dominant macular dystrophies including Best disease, adult onset vitelliform macular dystrophy and autosomal dominant vitreo-retinochoroidopathy. The protein is integral to the membrane and is likely involved in Ca^2+^-dependent transport of chloride ions across cellular membranes. Bestrophin 1 together with its three homologues forms a phylogenetically highly conserved family of proteins.

**Results:**

A bioinformatics study was performed to investigate the phylogenetic relationship among the bestrophin family members and to statistically evaluate sequence conservation and functional divergence. Phylogenetic tree assembly with all available eukaryotic bestrophin sequences suggests gene duplication events in the lineage leading to the vertebrates. A common N-terminal topology which includes four highly conserved transmembrane domains is shared by the members of the four paralogous groups of vertebrate bestrophins and has been constrained by purifying selection. Pairwise comparison shows that altered functional constraints have occurred at specific amino acid positions after phylogenetic diversification of the paralogues. Most notably, significant functional divergence was found between bestrophin 4 and the other family members, as well as between bestrophin 2 and bestrophin 3. Site-specific profiles were established by posterior probability analysis revealing significantly divergent clusters mainly in two hydrophilic loops and a region immediately adjacent to the last predicted transmembrane domain. Strikingly, codons 279 and 347 of human bestrophin 4 reveal high divergence when compared to the paralogous positions strongly indicating the functional importance of these residues for the bestrophin 4 protein. None of the functionally divergent amino acids were found to reside within obvious sequences patterns or motifs.

**Conclusion:**

Our study highlights the molecular evolution of the bestrophin family of transmembrane proteins and indicates amino acid residues likely relevant for distinct functional properties of the paralogues. These findings may provide a starting point for further experimental verifications.

## Background

The bestrophins are a phylogenetically conserved family of integral membrane proteins initially identified in *Caenorhabditis elegans *[1]. Homologous sequences are found in animals, fungi, and prokaryotes, but not in protozoans or plants [2]. Conservation is mainly restricted to the N-terminal 350–400 amino acids with an invariant motif arginine-phenylalanine-proline (RFP) of unknown functional properties.

The first human bestrophin cloned, bestrophin 1, is encoded by the vitelliforme macular dystrophy type 2 (VMD2) gene on chromosome 11q13 and was shown to be associated with Best macular dystrophy (BMD, OMIM #153700) also known as Best disease [3,4]. Subsequently, mutations in this gene were also found to cause adult onset vitelliform macular dystrophy (AVMD, OMIM #608161) and autosomal dominant vitreo-retinochoroidopathy (ADVIRC, OMIM #193220). The currently known 106 disease-causing mutations [5] are associated with a dominant pattern of inheritance with the majority being missense mutations located in four clusters near the RFP motif and the predicted transmembrane domains (TMDs) [6].

Based on immunocytochemical studies in macaque, porcine and human eyes, bestrophin 1 was shown to localize to the basolateral plasma membrane of the retinal pigment epithelium (RPE) [7,8]. In addition, bestrophin 1 is broadly expressed in other epithelia including the intestine and the lung [9], whereas bestrophin 2 expression appears confined to the olfactory epithelium [10]. On the functional level, whole cell patch clamp experiments suggest that the bestrophins act as Ca^2+^-dependent transporters of chloride ions across epithelial borders [11-16]. Functional proteins likely exist as multimeric complexes eventually by forming homo- or heteromeric associations between bestrophin family members.

Uncertainty exists as to the specific function of bestrophin 1. As an alternative to its activity as a chloride channel, bestrophin 1 may act as an accessory protein in the regulation of voltage-gated calcium channels [17]. In addition, bestrophin 1 could be involved in the volume sensitivity of RPE cells during phagocytosis of photoreceptor outer segments [11]. Consequently, distinct functional aspects of bestrophin 1 may contribute to RPE dysfunction and thus may explain the variable phenotypes associated with a mutant protein.

Phylogenetic studies of protein families can be a valuable tool to determine conserved but also divergent regions, potentially leading to functional predictions [18]. In this study we elucidated the evolutionary history of the bestrophins and identified structural and putative functional motifs of the bestrophin protein family by a comprehensive bioinformatics/phylogenetic approach. This has led us to predict distinct amino acid residues that may be of importance in the functional divergence of the bestrophin paralogues.

## Results

### Phylogenetic analysis of the bestrophin family

We first retrieved the available bestrophin sequences from the currently sequenced genomes. Querying major databases and unfinished genomes with the full-length amino acid sequences from the four human bestrophin paralogues identified 173 homologous proteins in vertebrates (mammals, birds, amphibians and fishes), urochordates (sea squirt), and invertebrates (insects, nematodes) (see Additional file 1). While *C. elegans *reveals a large number of bestrophins (n = 26), most other organisms harbour three or four family members. In each of the recently sequenced genomes of the urochordata *Ciona inestinalis *and *Ciona savigny*, the closest relatives of the craniates, we identified only a single bestrophin sequence strongly suggesting that gene duplication events may have occurred in the lineage leading to the vertebrates.

**Figure 1:**
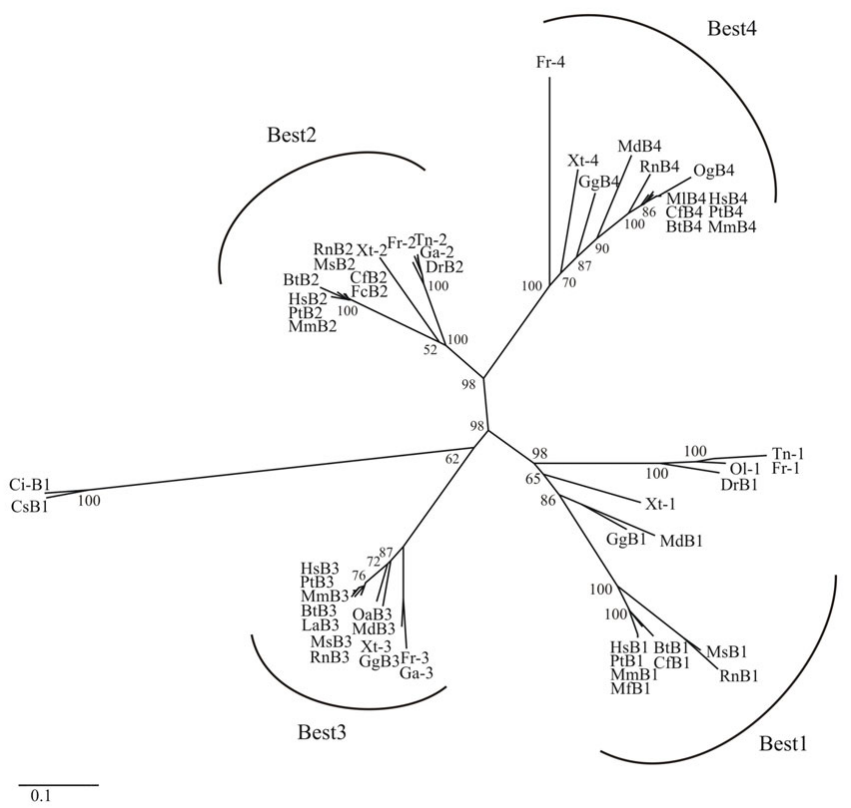
**Phylogenetic tree of 53 homologues of vertebrate bestrophins**. The phylogenetic tree of the bestrophin protein family was inferred by the neighbor-joining method (NJ) method applying gamma corrected distances. Urochordata (*Ciona intestinalis *[Ci] and *Ciona savigny *[Cs]) were used as out-group. The support for each phylogenetic group was tested with 1000 bootstrap pseudoreplicates. Protein sequences are given in Table 1.

Our further study focused on vertebrate bestrophins. After exclusion of unfinished and partial protein sequences, a phylogenetic tree with 53 selected bestrophins (Table 1) was constructed by the neighbour-joining (NJ) method based on a gamma corrected Jones-Taylor-Thornton (JTT) distance matrix [19,20] (Figure 1). Accordingly, the vertebrate bestrophins can unambiguously be separated into distinct clusters. With the urochordata as outgroup, the vertebrate bestrophins form four separate monophyletic groups, all with high bootstrap support indicating that the formation of the paralogous subfamilies occurred before the divergence of individual species (Figure 1). The phylogenetic branches of bestrophins 1 and 3 separated considerably earlier in evolution than bestrophin subgroups 2 and 4, partially explaining the level of sequence conservation between each of the two subfamilies. The high level of sequence identity within a subfamily suggests evolutionarily conserved functions. Overall, the data indicate that the bestrophin subfamilies have evolved by gene duplication events, in good agreement with previous findings demonstrating that large-scale gene duplications have occurred during chordate evolution [21,22].

**Table 1:** Vertebrate bestrophin homologues (n = 53) used for phylogenetic analysis

No.	Protein Name	Short name	Organism	Taxonomy	Amino acids	Gene bank identifier	cDNA Acc. No.
1	Bestrophin 1	HsB1	Homo sapiens	Mammalia	585	NP_004174	AF073501
2	Bestrophin 2	HsB2			509	AAR99655	AY515705
3	Bestrophin 3	HsB3			668	NP_116124	NM_032735
4	Bestrophin 4	HsB4			473	NP_695006	NM_153274
5	Bestrophin 1	PtB1	Pan troglodytes	Mammalia	585	XP_001151529	XM_001151529
6	Bestrophin 2	PtB2			509	XP_512414	XM_512414
7	Bestrophin 3	PtB3			668	XP_522466	XM_522466
8	Bestrophin 4	PtB4			473	XP_524571	XM_524571
9	Bestrophin 1	MmB1	Macaca mulatta	Mammalia	585	XP_001116583	XM_001116583
10	Bestrophin 2	MmB2			509	XP_001108800	XM_001108800
11	Bestrophin 3	MmB3			669	XP_001117392	XM_001117392
12	Bestrophin 4	MmB4			473	XP_001098771	XM_001098771
13	Bestrophin 1	MfB1	Macaca fascicularis	Mammalia	585	AAQ56049	AY357925
14	Bestrophin 1	BtB1	Bos taurus	Mammalia	589	XP_585778	XM_585778
15	Bestrophin 2	BtB2			528	XP_607911	XM_607911
16	Bestrophin 3	BtB3			369	XP_613863	XM_613863
17	Bestrophin 4	BtB4			467	XP_587691	XM_587691
18	Bestrophin 3	LaB3	Loxodonta africana	Mammalia	674	ENSLAFP00000000778	ENSLAFT00000000928
19	Bestrophin 2	FcB2	Felis catus	Mammalia	488	ENSFCAP00000000436	ENSFCAT00000000470
20	Bestrophin 1	CfB1	Canis familiaris	Mammalia	693	XP_540912	XM_540912
21	Bestrophin 2	CfB2			509	XP_542045	XM_542045
22	Bestrophin 4	CfB4			437	XP_539638	XM_539638
23	Bestrophin 1	MsB1	Mus musculus	Mammalia	551	NP_036043	NM_011913
24	Bestrophin 2	MsB2			508	AAS09923	AY450428
25	Bestrophin 3	MsB3			669	NP_001007584	NM_001007583
26	Bestrophin 1	RnB1	Rattus norvegicus	Mammalia	550	NP_001011940	NM_001011940
27	Bestrophin 2	RnB2			508	XP_001070841	XM_001070841
28	Bestrophin 3	RnB3			672	XP_235161	XM_235161
29	Bestrophin 4	RnB4			454	XP_001066317	XM_001066317
30	Bestrophin 1	MdB1	Monodelphis domestica	Mammalia	467	XP_001363751	XM_001363714
31	Bestrophin 3	MdB3			676	XP_001369557	XM_001369520
32	Bestrophin 4	MdB4			487	XP_001376079	XM_001376042
33	Bestrophin 4	OgB4	Otolemur garnettii	Mammalia	471	ENSOGAP00000006199	ENSOGAT00000006927
34	Bestrophin 4	MlB4	Myotis lucifugus	Mammalia	473	ENSMLUP00000001435	ENSMLUT00000001564
35	Bestrophin 3	OaB3	Ornithorhynchus anatinus		675	ENSOANP00000009484	ENSOANT00000009486
36	Bestrophin 1	GgB1	Gallus gallus	Aves	762	XP_421055	XM_421055
37	Bestrophin 3	GgB3			669	XP_416091	XM_416091
38	Bestrophin 4	GgB4			488	XP_001234941	XM_001234940
39	Bestrophin 1	Xt-1	Xenopus tropicalis	Amphibia	419	ENSXETP00000014724	ENSXETT00000014724
40	Bestrophin 2	Xt-2			510	NP_988974	NM_203643
41	Bestrophin 3	Xt-3			366	ENSXETP00000002984	ENSXETT00000002984
42	Bestrophin 4	Xt-4			367	ENSXETP00000029791	ENSXETT00000029791
43	Bestrophin 1	Tr-1	Takifugu rubripes	Actinopterygii	369	SINFRUP00000141703	SINFRUT00000141703
44	Bestrophin 2	Tr-2			431	SINFRUP00000151123	SINFRUT00000151123
45	Bestrophin 3	Tr-3			407	SINFRUP00000181928	SINFRUT00000182495
46	Bestrophin 4	Tr-4			370	SINFRUP00000134584	SINFRUT00000134584
47	Bestrophin 1	DrB1	Danio rerio	Actinopterygii	367	XP_689098	XM_684006
48	Bestrophin 2	DrB2			589	XP_695597	XM_690505
49	Bestrophin 1	Tn-1	Tetraodon igrovoridis	Actinopterygii	572	CAG08784	CAAE01015000
50	Bestrophin 2	Tn-2			431	CAG03298	CAAE01014712
51	Bestrophin 2	Ga-2	Gasterosteus culeatus	Actinopterygii	368	ENSGACP00000025248	ENSGACT00000025297
52	Bestrophin 3	Ga-3			367	ENSGACP00000019354	ENSGACT00000019392
53	Bestrophin 1	Ol-1	Oryzias latipes	Actinopterygii	454	ENSORLP00000007714	ENSORLT00000007715

### N-terminal topology of the vertebrate bestrophins

**Figure 2:**
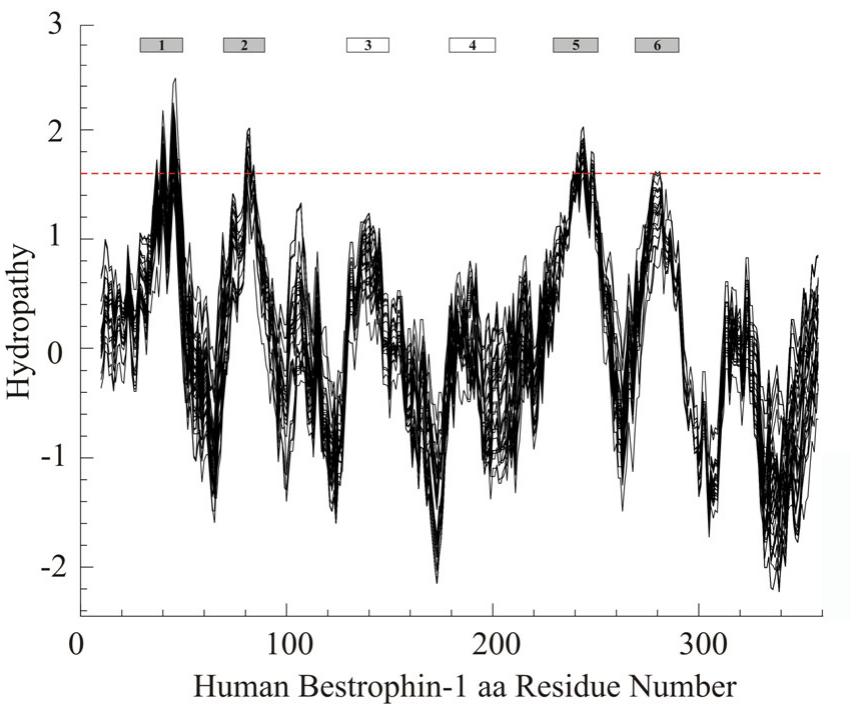
**Average hydropathy plot of 53 homologues of vertebrate bestrophins**. Hydropathy plot was generated from 53 protein sequences as given in Table 1. A threshold value of 1.6 is marked by the dotted line. Positive values indicate hydrophobic regions. Putative transmembrane domains were determined by the TOPPRED II algorithm and are depicted as gray and white boxes on the top.

We next analyzed whether the strong sequence conservation between the N-terminal regions of the vertebrate bestrophins also predicts a common topology. Putative transmembrane domains and hydrophobic regions were modelled *in silico *(Figure 2). Hydropathy plotting of 53 bestrophins (orthologues *and *paralogues, Table 1) revealed six hydrophobic regions including four putative transmembrane domains with hydropathy values reaching or above the threshold of 1.6 (Figure 2). These four hydrophobic peaks are strongly conserved in the vertebrate bestrophins suggesting membrane insertion of all bestrophin family members similar to bestrophin 1 [23].

### Variable selective pressures among amino acid sites

To analyze positive or negative selection of specific amino acid regions within the full-length protein sequences of mammalian bestrophins, substitution rate ratios of non-synonymous (dN) versus synonymous (dS) mutations (dN/dS or ω) were calculated for vertebrate bestrophins as given in Table 1. A dN/dS value <1 is indicative of purifying selection acting against amino acid changes, whereas dN/dS values >1 indicate an excess of amino acid changes suggesting adaptive evolution [24,25]. Amino acids in a protein sequence are expected to be under different selective pressure and to have different underlying dN/dS ratios. Table 2 shows that the ratio values for the vertebrate bestrophins are substantially lower than 1 suggesting that the N-termini of mammalian bestrophins within each subgroup were under strong purifying selection pressure. In order to test for positive selection at individual amino acid codons, the site-specific models implemented in codeml were used. Likelihood rate tests (LRTs) were performed between model M0 (one ratio) and M3 (discrete), M1a (nearly neutral) and M2a (positive selection), and M7 (beta) and M8 (beta and ω) (Table 2). The selection model (M2a) does not suggest presence of positively selected sites (P = 1). M0 was rejected when compared to M3 (P < 0.001), although no positively selected sites were detected. This could be explained by the fact that the majority of the protein is subjected to constant purifying selection, while a few sites undergo positive selection [26]. From the three models which allow for selection to be tested (M2a, M3 and M8), only M8 was significantly favoured over M7 (P < 0.001) and detected 44 sites at the 95% level (Table 2). This provides evidence for the Darwinian selection in vertebrate bestrophins.

**Table 2:** Likelihood values and parameter estimates for the vertebrate bestrophin genes

Model	**dN/dS**^a^	**Estimates of parameters**^b^	l	**Positively Selected Sites**^c^
M0 (one-ratio)	0.053	ω = 0.053	-22547.97	not allowed
M1a (nearly neutral)	0.084	p_0 _= 0.964, (p_1 _= 0.036) (ω_0 _= 0.050), (ω_1 _= 1)	-22443.90	not allowed
M2a (positive selection)	0.084	p_0 _= 0.964, p_1 _= 0.002, (p_2 _= 0.033) (ω_0 _= 0.050), (ω_1 _= 1), ω_2 _= 1	-22443.90	not found
M3 (discrete)	0.058	p_0= _0.529, p_1= _0.355, (p_2 _= 0.115) ω_0 _= 0.011, ω_1 _= 0.073, ω_2 _= 0.230	-22016.13	not found
M7 (beta)	0.063	p = 0.598, q = 8.411	-22007.24	not allowed
M8 (beta&ω)	0.220	p_0_= 1, (p_1 _= 0) p = 0.685, q = 1.988, ω = 4.997	-19662.03	8, 25, **40**, **41**, 42, 44, 45*, 48, **49**, 52*, 53, **55**, **56**, **57**, 60*, 61*, 67, 71, 95, 108, 113, **117**, **120**, 154, 159, 165*, 170, 173, 175, 193, **197**, 207*, 209*, 216, 219, **261**, 263*, 265*, 331*, 340, **341**, 342, 344, 354*

^a ^The dN/dS ratio is an average over all sites of bestrophin gene alignments.

^b ^Parameters in parentheses are not free parameters.

^c ^Numbering of amino acid residues corresponds to human bestrophin 1. Positive selection sites with posterior probabilities > 95% are shown in bold.

* The amino acid residues depicted with an asterisk were also found to be implicated in the functional divergence between bestrophin paralogues (see Table 4).

### Analysis of functional divergence

To further investigate whether amino acid substitutions in the highly conserved N-termini of the bestrophins could have caused adaptive functional diversification, amino acid residues 1–367 from 53 vertebrate bestrophins (Table 1) were used for posterior analysis by the DIVERGE program algorithms [27,28]. Pairwise comparisons of paralogous bestrophins from subfamilies 1 to 4 were carried out and the rate of amino acid evolution at each sequence position was estimated. This identified residues potentially responsible for functional divergence. The results are given as a coefficient of functional divergence (θ) with standard errors and significance levels (Table 3). Significant functional divergence was detected between bestrophin 2 and 3 (P_corr _= 6.3 × 10^-9^) as well as between bestrophin 4 and the other bestrophin subgroups (P_corr _≤ 8.9 × 10^-8^).

**Table 3:** Functional divergence estimated in 53 vertebrate bestrophin paralogues

Comparison	**θ**^a^	**SE**^b^**(θ)**	LRT^c ^(θ)	**P**^d^	**Probability cutoff**^e^
Best1 vs Best2	0.097	0.076	1.640	0.024541	-
Best1 vs Best3	0.142	0.043	10.762	0.000488	-
Best1 vs Best4	0.217	0.039	30.099	8.88557e-8	0.70
Best2 vs Best3	0.380	0.073	27.152	6.32074e-9	0.80
Best2 vs Best4	0.320	0.075	18.120	9.855e-10	0.64
Best3 vs Best4	0.260	0.059	19.338	9.477e-10	0.70

^a ^θ is the coefficient of functional divergence.

^b ^SE, standard error.

^c ^LRT (θ) is a likelihood ratio test.

^d ^significance level (P^c^) calculated by the method of Fisher's transformation and corrected by Bonferroni.

^e ^Probability cut-off is the minimal posterior probaility for amino acids causing functional divergence.

**Figure 3:**
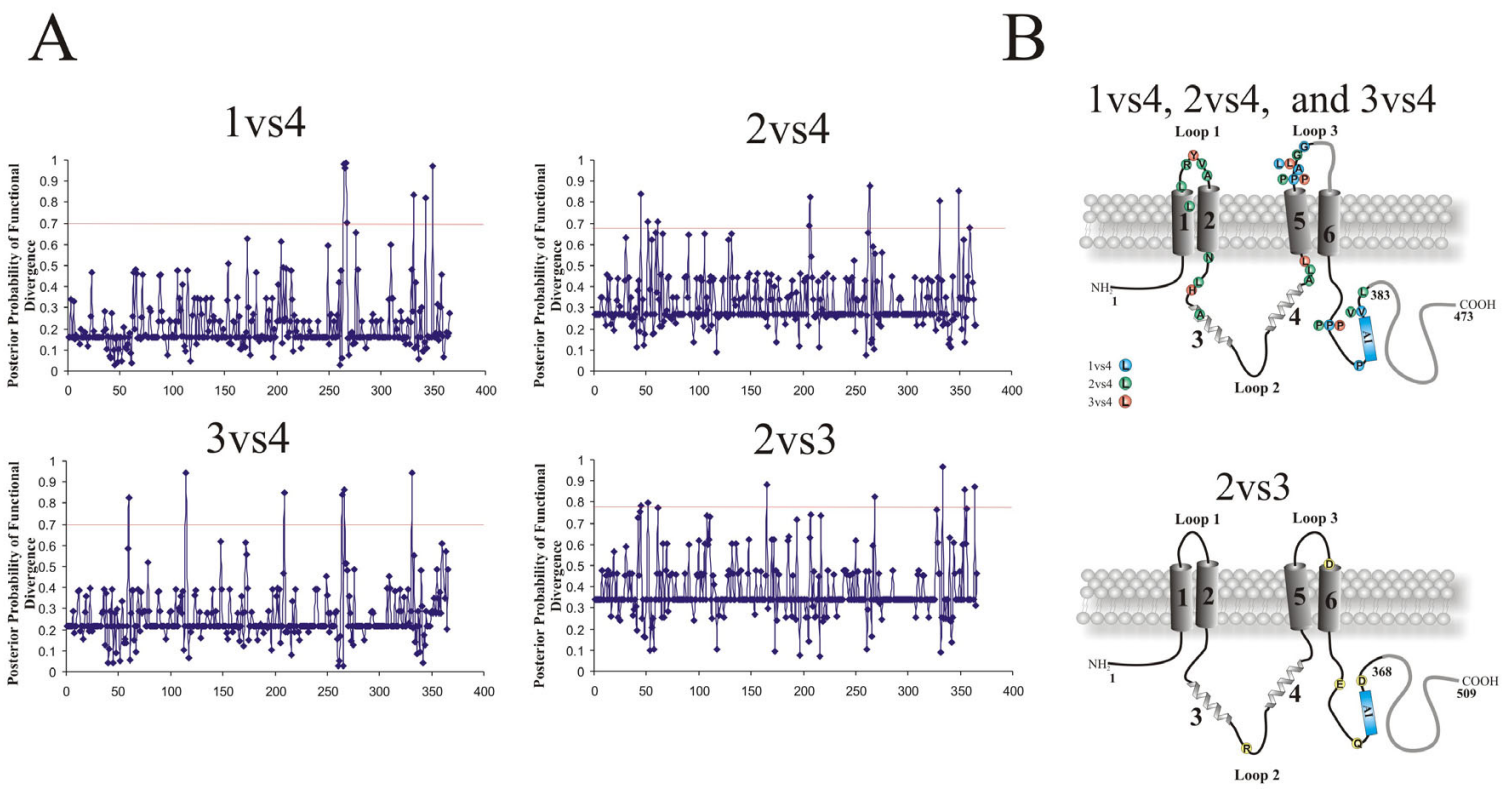
**Site specific profiles for evolutionary rate changes in the vertebrate bestrophin protein family**. **A**, The posterior probabilities of functional divergence for vertebrate bestrophins 1 to 4 were obtained with Diverge [27]. Individual cut-off values for each comparison are marked with red horizontal lines. **B**, Residues with predicted functional divergence between bestrophin subfamilies are mapped onto the membrane topology models of bestrophin 4 (top) and bestrophin 2 (bottom). Divergent amino acids are shown and listed in Table 4. In the model drawing, residues excluded from the analysis are depicted in gray.

Next, single amino acid residues responsible for the functional divergence were predicted based on site-specific profiles (Figure 3A) in combination with suitable cut-off-values derived from the posterior probability of each comparison (Table 3). Residues predicted to be functionally divergent in bestrophin, were mapped onto topology models of human bestrophins 2 and 4, respectively (Figure 3b). The predicted functional sites are not equally distributed throughout the respective bestrophin, but instead are clustered in the hydrophilic loops between predicted transmembrane domains 1 and 2 as well as 5 and 6 and immediately C-terminal to transmembrane domain 6 (Figure 3B, see Additional file 2, and Table 4). Despite the high global sequence identity of mammalian bestrophins 2 and 3, functionally divergent amino acids were also identified between these bestrophins. For example, at position 264 (numbered according to bestrophin 1) the tyrosine residue is completely conserved within bestrophin 1, 2 and 3 paralogues but highly divergent in bestrophin 4 paralogues. Similarly, residue 331 (numbered according to bestrophin 1) is highly divergent in bestrophin 1, 2 and 3 paralogues but strictly conserved in bestrophins 4 (Figure 3B, see Additional file 2, and Table 4). We thus speculate that these two sites may be of functional importance for the respective bestrophin subfamilies. In addition, 11 out of 44 positively selected sites detected by LRT in codeml were also found to be functionally divergent between bestrophin paralogues (marked by asterisk in Table 4).

**Table 4:** Amino acid changes associated with the divergence of human bestrophins

Comparison	**Codon position in human Best1**^a^	**Codon position and amino acid residue**^b^	**Codon position and amino acid residue**^b^	**Protein domain**^c^
Best1 vs. Best4		in vertebrate Best1	in vertebrate Best4	

	264	264: Y, conserved	279: P, Q, S, L, E, G or Y	Loop 3
	265*	265: P, conserved	280: A, T, Q, E or K	Loop 3
	266	266: G, conserved	281: L, A, V, P, K, E or D	Loop 3
	267	267: H, conserved	282: G, W, P or H	Loop 3
	331*	331: R, P, V, I, L or M	346: P, conserved	C-terminus
	343	343: P, conserved	358: P, A, F or V	C-terminus
	349	349: A, conserved	364: V, I, L, P, Q or A	C-terminus

Best2 vs Best4		in vertebrate Best2	in vertebrate Best4	

	45*	45: A, conserved	45: V, L, M, F or T	TMD2
	52*	52: F, conserved	52: L, K, R, C or F	Loop 1
	59	59: K, conserved	59: R, K or Q	Loop 1
	61*	61: Y, conserved	61: V, I, L, M or E	Loop 1
	66	66: V, A or S	66: A, conserved	Loop 1
	91	91: N or H	91: N, conserved	Loop 2
	106	106: L or V	106: L, conserved	Loop 2
	132	132: A, S or C	132: A, conserved	Loop 2
	206	206: A, T or S	206: A, conserved	Loop 2
	207*	207: L, F or Y	207: L, conserved	Loop 2
	263*	263: G, conserved	263: G, E, A or P	Loop 3
	264	264: Y, conserved	279: P, E, Q, G, S or Y	Loop 3
	331	331: V, M or P	346: P, conserved	C-terminus
	349	349: A, conserved	364: V, P, Q, L or A	C-terminus

Best3 vs Best4		in vertebrate Best3	in vertebrate Best4	

	60*	60: R, conserved	60: Y, H, R, S or D	Loop 1
	115	115: H, Q, R or L	115: H, conserved	Loop 2
	209*	209: S, T, M or N	209: L, conserved	Loop 2
	264	264: Y, conserved	279: P, Q, S, L, E, G or Y	Loop 3
	266	266: G, conserved	281: L, V, E, P, K, D or A	Loop 3
	331	331: K or R	346: P, conserved	C-terminus

Best2 vs Best3		in vertebrate Best2	in vertebrate Best3	

	165*	165: R, conserved	165: T, A, P, S or R	Loop 2
	268	268: D, T, S or N	268: D, conserved	Loop 3
	333	333: E, conserved	333: K, R, E, A, N or T	C-terminus
	354*	354: Q, L, D or V	354: Y, conserved	C-terminus, AI domain
	365	366: D, conserved	365: Q, D, E or H	C-terminus, AI domain

We finally scanned the predicted divergent amino acids and surrounding sequences in bestrophins 2 and 4 for the presence of domains and functional sequence patterns with MotifScan and InterProScan. Identity to known protein motifs, except for several short sequence tags including putative phosphorylation sites, could not be detected.

## Discussion

Our phylogenetic analysis suggests that the bestrophins originated by duplication and divergence of a common protein at the base of the eukaryotic tree, some 700 Myr ago. Analysis of the bestrophins from a phylogenetic perspective may provide the basis for understanding the functional diversity within this conserved protein family. Both small-scale and large-scale gene duplications are known to contribute to the complexity of eukaryotic organisms [29,30]. The presence of bestrophins in prokaryotes [2] and their occurrence in phylogenetically distant eukaryotic species such as insects and mammals highlight their general functional importance.

High levels of sequence homology were generally found between the N-terminal regions of the bestrophins, while the C-termini differ substantially particularly between paralogues. At the N-terminal sequences, vertebrate bestrophins show nearly identical hydrophobicity plots with four major and two minor hydrophobic peaks, indicating that they share a highly similar membrane topology [16,23]. The hydrophobic regions with high sequence conservation also seem to contribute to oligomerization [15,31] and the second putative transmembrane domain is thought to be especially important for channel function [13,14,32].

Our data and the study by Hagen et al. [2] demonstrate that the vertebrate bestrophins cluster in organismal groups, i.e. the homologues are more related to each other from different species than to their species-specific paralogues. Whenever high quality genome sequences are available (e.g. human, chimp, dog, rat, and fugu), four bestrophin paralogues can be identified. This strongly argues for a high phylogenetic conservation of the bestrophin subfamilies. Missing paralogues in some species may be due to currently incomplete genomic sequences. One exception may be X. laevis, for which a high quality genome sequence has already been released but still this species reveals only three copies of bestrophins, interestingly all highly similar to bestrophin 2. However, as a tetraploid species, X. laevis appears to have undergone unique evolutionary changes. Another exception may be the murine bestrophin 4 (gene name: VMD2-L2) which our group has previously shown to represent a functionless pseudogene [33].

In the vertebrate phylogeny, bestrophins 1 and 3 have separated and diverged at a higher rate than bestrophins 2 and 4. To estimate the selection forces behind this we calculated the substitution rate ratios for the four subgroups. Calculating dN/dS ratios across the entire length of the N-terminal amino acid sequences (aa1-367), we could not, however, find an indication for positive selection. In contrast, the low dN/dS values imply that a strong purifying selection may lead to the high sequence conservation observed. Examples such as the chaperonins [24] show that positive selection reaches dN/dS values well above 1 for a high selective pressure to fix amino acid substitutions in evolution. Nevertheless, we detected positive selection at several amino acid sites located predominantly in the hydrophilic regions of bestrophin. Testing several models only one (M7vsM8) suggested positively selected sites with high posterior probabilities. Another comparison (M0 vs M3), although significant failed to identify positively selected residues with high posterior probability. This points to insufficient information from the alignments or, alternatively, may be explained by the fact that purifying selection in conserved regions could have masked single diversification signals.

Posterior probability analysis for pairwise comparisons of bestrophin paralogues identified significant functional divergence between bestrophin 2 and bestrophin 3 as well as between bestrophin 4 and its remaining paralogues. Generally, the specific amino acid substitutions in the bestrophin paralogues suggest an optimization of protein function, and/or subfunctionalization of the gene duplicates. Eleven out of the 44 positively selected sites were also found to be functionally divergent between bestrophins. The most prominent finding of the posterior probability analysis is that bestrophin 4 diverged significantly during evolution in a number of amino acids from its paralogues. As shown previously [34], bestrophin 4 can be activated by free Ca^2+ ^on the cytoplasmic side although the kinetics of the activation/deactivation is much slower than for typical Ca^2+^-activated chloride currents [35]. Furthermore, heterologously expressed human bestrophin 4 shows very slow voltage-dependant current relaxations, in contrast to the lack of voltage-dependent current relaxations by human bestrophin 1 and 2. These distinctive properties of bestrophin 4 could be attributable to the additional 15 unique amino acids within the extracellular loop between putative transmembrane domain 5 and 6. This loop contains three positively charged lysine residues (K266, K269 and K272) within a proline rich region (P267, P273, P277 and P279). It can be speculated that the function of the divergent P279 residue could be to maintain structural or conformational stability of the loop region.

Human bestrophin 1 and 3 have considerably longer amino acid sequences compared to bestrophin 2 and 4 (585 and 668 vs 509 and 473 aa). Human and mouse bestrophin 3, heterologously expressed in HEK-293 cells, produce no chloride currents in a physiological range [16,36], in contrast for example to mouse bestrophin 2 [13,14]. Such a functional divergence has been explained to be mediated by an auto-inhibitory (AI) domain composed of seven critical residues _356_IPSFLGS_362 _present in mouse bestrophin 3 [36]. Interestingly 2 out of 5 amino acid residues implicated in the functional divergence between bestrophin 2 and 3 are located in close proximity to this highly conserved region (Q354 and D366 in bestrophin 2, respectively).

## Conclusion

This study addresses the evolutionary history of the bestrophin protein family. The precise functional properties of the four family members are still unclear but may comprise activities as Ca^2+^-activated chloride channels or aspects of Ca^2+ ^channel regulation. Functional divergence between the paralogous bestrophins suggests that the bestrophin family members have evolved different functional properties after gene duplication events which have occurred early in the vertebrate lineage. This is most evident for bestrophin 4 where a number of experimental studies provide further support for the *in silico *data. Our study also demonstrates that amino acids critical for functional divergence are located in regions of the proteins that are likely accessible to soluble ligands, supporting the biochemical and physiological significance of this prediction.

## Methods

### Data collection

PSI-BLAST and TBLASTN searches with protein sequences of the four human bestrophins were performed in protein databases or unfinished genome sequencing projects at NCBI [37], ENSEMBL [38], the Sanger Institute [39], UCSC Genome Bioinformatics Group [40], and the Joint Genome Institute [41]. Proteins identified by the BLAST search algorithms were considered as potential homologues when amino acid identity was above 25% over a stretch of = 200 amino acids. After removal of expressed sequence tags, splice variants and redundant sequences, the initial data set included 173 distinct sequences from 43 species (see Additional file 1).

### Sequence alignment and phylogenetic tree reconstruction

Amino acid sequences were aligned with CLUSTAL W (version 1.82) [42] and alignments were refined manually in Genedoc [43]. Incomplete sequences, and highly divergent regions or gaps resulting in uncertain alignments were excluded from the analysis. The final data set includes a total of 53 sequences from 21 vertebrate species (Table 1). A total of 367 N-terminal amino acids were aligned for further studies. Accurate nucleotide alignments were obtained with PAL2NAL [44], a program which constructs multiple codon alignments from matching protein sequences. ProtTest v1.4 [45], implementing the Akaike Information criterion (AIC) was used to estimate the most appropriate model of amino acid substitution for tree building analyses. The best fit model of protein evolution for the bestrophin protein family according to ProtTest corresponds to a JTT+I+G+F model [19].

Tree reconstructions were done by the Neighbor-joining method (NJ) [20] from the protein alignment done in the MEGA v3.1 software package, with the gamma distribution model implemented to account for heterogeneity among sites [46], and rooted with the bestrophins from Urohordata. The shape parameter of the gamma distribution (α) was estimated using baseml from the PAMLv4.0 [47] to be α = 0.51. Support for each phylogenetic group was tested using 1,000 bootstrap pseudoreplicates. Tree topology assessed by maximum parsimony (using Mega v3.1), was substantial similar with the NJ tree.

### Topological structure prediction

Topological structure prediction was done with the TOPPRED II software [48], which is based on the Kyte and Doolittle algorithm [49]. The average hydrophobicity values of putative transmembrane domains of 20–23 amino acid residues were calculated according the Eisenberg scale [50]. An average hydropathy plot of 53 bestrophin-related protein sequences was generated by the PEPWINDOWALL program with a window of 19 amino acids [49].

### Positive selection assessment

DNA sequences and related multiple protein sequence alignments were submitted to the PAL2NAL web server [51] which converts a multiple sequence alignment of proteins and the corresponding DNA sequences into a codon alignment. The resulting codon alignments and NJ tree were used in the program codeml from the PAMLv4.0 software package [47] to calculate the dN/dS (or ω) ratio for each site and to test different evolutionary models. The site specific models recommended by Anisimova et al. [26] were tested: Model M0 (one ratio), M1a (nearly neutral), M2a (positive selection), M3 (discrete), M7 (beta) and M8 (beta+ ω). Model M0 assumed a constant ω-ratio, while in models M1a and M2a ω-ratio is estimated from the date (0 < ω_0 _< 1) while ω_1 _= 1 is fixed. M7 and M8 assume a β-distribution for the ω-value between 0 and 1. Models M2a, M3, and M8 allow the occurrence of positively selected sites (ω >1). Subsequent likelihood rate comparisons of M0 with M3, M1a with M2a, and M7 with M8, respectively, were performed to test which model fits the data significantly better. Twice the difference in log likelihood between the models is compared with a chi-square distribution with n degrees of freedom, n being the difference between the numbers of parameters of the two models.

### Functional divergence and detection of amino acids critical for altered functional constraints

Bestrophin sequence duplication events were tested for type I functional divergence [52] based on the method by Gu et al [27]. The analysis was carried out with Diverge (version 2.0) [28]. This method is based on maximum likelihood procedures to estimate significant changes in the rate of evolution after the emergence of two paralogous sequences. Type I sites represent amino acid residues conserved in one subfamily but highly variable in another, implying that these residues have been subjected to different functional constraints. A set of 53 protein sequences was included in the study (Table 1, see Additional file 2). Due to gaps and a shorter length of bestrophin 2 and 4, a total of 15 residues from human bestrophin 4 (codons 264–278) and one (codon 356) from human bestrophin 2 were excluded from the analysis. Consequently, each bestrophin paralogue was restricted to 367 amino acid residues. A new NJ tree was constructed within Diverge with Poisson distance and re-rooted. The coefficient of functional divergence (θ) and the posterior probability for the functional divergence were calculated for each position in the alignment. To detect amino acid residues reflecting functional divergence, bestrophin subfamilies were pair-wise compared to each other. The cut-off value for the posterior probability was determined by consecutively eliminating the highest scoring residues from the alignment until the coefficient of functional divergence dropped to zero.

## Authors' contributions

VMM performed the bioinformatics analyses including all sequence alignments and statistical evaluations. He drafted the manuscript. TL was involved in conceptual discussions for the entire study and made major revisions to the manuscript. RS and KK contributed to the study concept and corrected the manuscript. BHFW participated in conception and design of the study and revised and finalized the manuscript critically. All authors read and approved the final manuscript.

## Supplementary Material

Additional file 1 List of 173 bestrophin homologues identified in public databases. This table lists the molecular features of all 173 bestrophin homologues identified in public databases. Click here for file

Additional file 2 Multiple sequence alignment of vertebrate bestrophins used for calculations of functional divergence. Protein sequences of 53 homologues of vertebrate bestrophins were aligned using the ClustalW algorithm. Shown is the amino acid sequence alignment after removal of gaps and highly divergent regions. Numbering is given according to human bestrophin 1. Sites with predicted functional divergence between bestrophin subfamilies are highlighted by vertical yellow boxes. Click here for file
