# Supplementary material for: Molecular evolution and functional divergence of the bestrophin protein family
Source: BMC Evol Biol. 2008 Feb 28;8:72. doi: 10.1186/1471-2148-8-72 (PMC2292144; doi:10.1186/1471-2148-8-72)
Supplement: Additional file 1 — List of 173 bestrophin homologues identified in public databases. This table lists the molecular features of all 173 bestrophin homologues identified in public databases. [file 1471-2148-8-72-S1.doc]

**Additional File 2 List of 173 bestrophin homologues identified in public databases**

| **No.** | **Protein Name** | **Short name** | **Organism** | **Taxonomy** | **Amino acids** | **Gene bank identifier^1^** | **cDNA Acc. No.^1^** | **Functional divergence sequence** |
| --- | --- | --- | --- | --- | --- | --- | --- | --- |
| 1 | Bestrophin 1 | HsB1 | Homo sapiens | Mammalia | 585 | NP_004174 | AF073501 | + |
| 2 | Bestrophin 2 | HsB2 |  |  | 509 | AAR99655 | AY515705 | + |
| 3 | Bestrophin 3 | HsB3 |  |  | 668 | NP_116124 | NM_032735 | + |
| 4 | Bestrophin 4 | HsB4 |  |  | 473 | NP_695006 | NM_153274 | + |
| 5 | Bestrophin 1 | PtB1 | Pan troglodytes | Mammalia | 585 | XP_001151529 | XM_001151529 | + |
| 6 | Bestrophin 2 | PtB2 |  |  | 509 | XP_512414 | XM_512414 | + |
| 7 | Bestrophin 3 | PtB3 |  |  | 668 | XP_522466 | XM_522466 | + |
| 8 | Bestrophin 4 | PtB4 |  |  | 473 | XP_524571 | XM_524571 | + |
| 9 | Bestrophin 1 | MmB1 | Macaca mulatta | Mammalia | 585 | XP_001116583 | XM_001116583 | + |
| 10 | Bestrophin 2 | MmB2 |  |  | 509 | XP_001108800 | XM_001108800 | + |
| 11 | Bestrophin 3 | MmB3 |  |  | 669 | XP_001117392 | XM_001117392 | + |
| 12 | Bestrophin 4 | MmB4 |  |  | 473 | XP_001098771 | XM_001098771 | + |
| 13 | Bestrophin 1 | MfB1 | Macaca fascicularis | Mammalia | 585 | AAQ56049 | AY357925 | + |
| 14 | Bestrophin 1 | BtB1 | Bos taurus | Mammalia | 589 | XP_585778 | XM_585778 | + |
| 15 | Bestrophin 2 | BtB2 |  |  | 528 | XP_607911 | XM_607911 | + |
| 16 | Bestrophin 3 | BtB3 |  |  | 369 | XP_613863 | XM_613863 | + |
| 17 | Bestrophin 4 | BtB4 |  |  | 467 | XP_587691 | XM_587691 | + |
| 18 | Bestrophin 1 | LaB1 | Loxodonta africana | Mammalia | 585 | ENSLAFP00000004036* | ENSLAFT00000004820* |  |
| 19 | Bestrophin 3 | LaB3 |  |  | 674 | ENSLAFP00000000778 | ENSLAFT00000000928 | + |
| 20 | Bestrophin 4 | LaB4 |  |  | 398 | ENSLAFP00000002588* | ENSLAFT00000003113* |  |
| 21 | Bestrophin 1 | SsB1 | Sus scrofa | Mammalia | 428 | AAL40882* | AY064707* |  |
| 22 | Bestrophin 1 | FcB1 | Felis catus | Mammalia | 541 | ENSFCAP00000006844* | ENSFCAT00000007382* |  |
| 23 | Bestrophin 2 | FcB2 |  |  | 488 | ENSFCAP00000000436 | ENSFCAT00000000470 | + |
| 24 | Bestrophin 3 | FcB3 |  |  | 534 | ENSFCAP00000001496* | ENSFCAT00000001612* |  |
| 25 | Bestrophin 4 | FcB4 |  |  | 421 | ENSFCAP00000009253 | ENSFCAT00000009971 |  |
| 26 | Bestrophin 1 | CfB1 | Canis familiaris | Mammalia | 693 | XP_540912 | XM_540912 | + |
| 27 | Bestrophin 2 | CfB2 |  |  | 509 | XP_542045 | XM_542045 | + |
| 28 | Bestrophin 3 | CfB3 |  |  | 765 | XP_538279* | XM_538279* |  |
| 29 | Bestrophin 4 | CfB4 |  |  | 437 | XP_539638 | XM_539638 | + |
| 30 | Bestrophin 1 | MsB1 | Mus musculus | Mammalia | 551 | NP_036043 | NM_011913 | + |
| 31 | Bestrophin 2 | MsB2 |  |  | 508 | AAS09923 | AY450428 | + |
| 32 | Bestrophin 3 | MsB3 |  |  | 669 | NP_001007584 | NM_001007583 | + |
| 33 | Bestrophin 1 | RnB1 | Rattus norvegicus | Mammalia | 550 | NP_001011940 | NM_001011940 | + |
| 34 | Bestrophin 2 | RnB2 |  |  | 508 | XP_001070841 | XM_001070841 | + |
| 35 | Bestrophin 3 | RnB3 |  |  | 672 | XP_235161 | XM_235161 | + |
| 36 | Bestrophin 4 | RnB4 |  |  | 454 | XP_001066317 | XM_001066317 | + |
| 37 | Bestrophin 1 | CpB1 | Cavia porcellus | Mammalia | 541 | ENSCPOP00000005506* | ENSCPOT00000006169* |  |
| 38 | Bestrophin 2 | CpB2 |  |  | 452 | ENSCPOP00000000686* | ENSCPOT00000000773* |  |
| 39 | Bestrophin 3 | CpB3 |  |  | 612 | ENSCPOP00000001199* | ENSCPOT00000001340* |  |
| 40 | Bestrophin 3 | DnB3 | Dasypus novemcinctus | Mammalia | 673 | ENSDNOP00000007591* | ENSDNOT00000009799* |  |
| 41 | Bestrophin 4 | DnB4 |  |  | 471 | ENSDNOP00000007648* | ENSDNOT00000009872* |  |
| 42 | Bestrophin 1 | MdB1 | Monodelphis domestica | Mammalia | 467 | XP_001363751 | XM_001363714 | + |
| 43 | Bestrophin 2 | MdB2 |  |  | 459 | XP_001362839* | XM_001362802* |  |
| 44 | Bestrophin 3 | MdB3 |  |  | 676 | XP_001369557 | XM_001369520 | + |
| 45 | Bestrophin 4 | MdB4 |  |  | 487 | XP_001376079 | XM_001376042 | + |
| 46 | Bestrophin 2 | EtB2 | Echinops telfairi | Mammalia | 297 | ENSETEP00000004086* | ENSETET00000005016* |  |
| 47 | Bestrophin 3 | EtB3 |  |  | 660 | ENSETEP00000005622* | ENSETET00000006933* |  |
| 48 | Bestrophin 4 | EtB4 |  |  | 310 | ENSETEP00000003231 | ENSETET00000003940 |  |
| 49 | Bestrophin 1 | TbB1 | Tupaia belangeri | Mammalia | 592 | ENSTBEP00000007826* | ENSTBET00000009030* |  |
| 50 | Bestrophin 3 | TbB3 |  |  | 672 | ENSTBEP00000000335* | ENSTBET00000000388* |  |
| 51 | Bestrophin 4 | TbB4 |  |  | 464 | ENSTBEP00000000401* | ENSTBET00000000463* |  |
| 52 | Bestrophin 1 | StB1 | Spermoph. tridecemlineatus | Mammalia | 586 | ENSSTOP00000013004* | ENSSTOT00000014514* |  |
| 53 | Bestrophin 3 | StB3 |  |  | 654 | ENSSTOP00000013207* | ENSSTOT00000014743* |  |
| 54 | Bestrophin 4 | StB4 |  |  | 388 | ENSSTOP00000005663* | ENSSTOT00000006333* |  |
| 55 | Bestrophin 2 | OgB2 | Otolemur garnettii | Mammalia | 270 | ENSOGAP00000010924* | ENSOGAT00000012214* |  |
| 56 | Bestrophin 3 | OgB3 |  |  | 430 | ENSOGAP00000002064* | ENSOGAT00000002314* |  |
| 57 | Bestrophin 4 | OgB4 |  |  | 471 | ENSOGAP00000006199 | ENSOGAT00000006927 | + |
| 58 | Bestrophin 1 | OcB1 | Oryctolagus cuniculus | Mammalia | 552 | ENSOCUP00000012113* | ENSOCUT00000014094* |  |
| 59 | Bestrophin 4 | OcB4 |  |  | 416 | ENSOCUP00000010746* | ENSOCUT00000012482* |  |
| 60 | Bestrophin 1 | EeB1 | Erinaceus europaeus | Mammalia | 569 | ENSEEUP00000008004* | ENSEEUT00000008788* |  |
| 61 | Bestrophin 3 | EeB3 |  |  | 619 | ENSEEUP00000002330* | ENSEEUT00000002549* |  |
| 62 | Bestrophin 4 | EeB4 |  |  | 438 | ENSEEUP00000001670* | ENSEEUT00000001833* |  |
| 63 | Bestrophin 3 | MlB3 | Myotis lucifugus | Mammalia | 669 | ENSMLUP00000007674* | ENSMLUT00000008410* |  |
| 64 | Bestrophin 4 | MlB4 |  |  | 473 | ENSMLUP00000001435 | ENSMLUT00000001564 | + |
| 65 | Bestrophin 1 | OaB1 | Ornithorhynchus anatinus | Mammalia | 350 | ENSOANP00000008030* | ENSOANP00000008030* |  |
| 66 | Bestrophin 3 | OaB3 |  |  | 675 | ENSOANP00000009484 | ENSOANT00000009486 | + |
| 67 | Bestrophin 1 | GgB1 | Gallus gallus | Aves | 762 | XP_421055 | XM_421055 | + |
| 68 | Bestrophin 3 | GgB3 |  |  | 669 | XP_416091 | XM_416091 | + |
| 69 | Bestrophin 4 | GgB4 |  |  | 488 | XP_001234941 | XM_001234940 | + |
| 70 | Bestrophin 1 | Xl-1 | Xenopus laevis | Amphibia | 512 | AAH43854 | BC043854 |  |
| 71 | Bestrophin 2 | Xl-2 |  |  | 512 | AAH84229 | BC084229 |  |
| 72 | Bestrophin 3 | Xl-3 |  |  | 512 | AAP32200 | AY273826 |  |
| 73 | Bestrophin 1 | Xt-1 | Xenopus tropicalis | Amphibia | 419 | ENSXETP00000014724 | ENSXETT00000014724 | + |
| 74 | Bestrophin 2 | Xt-2 |  |  | 510 | NP_988974 | NM_203643 | + |
| 75 | Bestrophin 3 | Xt-3 |  |  | 366 | ENSXETP00000002984 | ENSXETT00000002984 | + |
| 76 | Bestrophin 4 | Xt-4 |  |  | 367 | ENSXETP00000029791 | ENSXETT00000029791 | + |
| 77 | Bestrophin 1 | Tr-1 | Takifugu rubripes | Actinopterygii | 563 | SINFRUP00000141703 | SINFRUT00000141703 | + |
| 78 | Bestrophin 2 | Tr-2 |  |  | 449 | SINFRUP00000151123 | SINFRUT00000151123 | + |
| 79 | Bestrophin 3 | Tr-3 |  |  | 627 | SINFRUP00000181928 | SINFRUT00000182495 | + |
| 80 | Bestrophin 4 | Tr-4 |  |  | 421 | SINFRUP00000134584 | SINFRUT00000134584 | + |
| 81 | Bestrophin 1 | DrB1 | Danio rerio | Actinopterygii | 367 | XP_689098 | XM_684006 | + |
| 82 | Bestrophin 2 | DrB2 |  |  | 589 | XP_695597 | XM_690505 | + |
| 83 | Bestrophin 4 | DrB4 |  |  | 426 | XP_692160* | XM_687068* |  |
| 84 | Bestrophin 1 | Tn-1 | Tetraodon nigrovoridis | Actinopterygii | 572 | CAG08784 | CAAE01015000 | + |
| 85 | Bestrophin 2 | Tn-2 |  |  | 431 | CAG03298 | CAAE01014712 | + |
| 86 | Bestrophin 3 | Tn-3 |  |  | 605 | CAF90002* | CAAE01007261* |  |
| 87 | Bestrophin 1 | Ga-1 | Gasterosteus aculeatus | Actinopterygii | 390 | ENSGACP00000007401* | ENSGACT00000007420* |  |
| 88 | Bestrophin 2 | Ga-2 |  |  | 368 | ENSGACP00000025248 | ENSGACT00000025297 | + |
| 89 | Bestrophin 3 | Ga-3 |  |  | 367 | ENSGACP00000019354 | ENSGACT00000019392 | + |
| 90 | Bestrophin 4 | Ga-4 |  |  | 193 | ENSGACP00000007406* | ENSGACT00000007425* |  |
| 91 | Bestrophin 1 | Ol-1 | Oryzias latipes | Actinopterygii | 454 | ENSORLP00000007714 | ENSORLT00000007715 | + |
| 92 | Bestrophin 2 | Ol-2 |  |  | 381 | ENSORLP00000021777 | ENSORLT00000021778 |  |
| 93 | Bestrophin 3 | Ol-3 |  |  | 127 | ENSORLP00000003967* | ENSORLT00000003968* |  |
| 94 |  | CiB1 | Ciona intestinalis | Ascidiacea | 366 | a | a |  |
| 95 |  | CsB1 | Ciona savigny | Ascidiacea | 394 | ENSCSAVP00000011171* | ENSCSAVG00000006529* |  |
| 96 |  | Ag-1 | Anopheles gambiae | Insecta | 594 | XP_558233 | XM_558233 |  |
| 97 |  | Ag-2 |  |  | 573 | XP_320767 | XM_320767 |  |
| 98 |  | Dp-1 | Drosophila pseudoobscura | Insecta | 522 | XP_001353161 | XM_001353125 |  |
| 99 |  | Dp-2 |  |  | 720 | XP_001358209 | XM_001358172 |  |
| 100 |  | Dp-3 |  |  | 446 | XP_001353162* | XM_001353126* |  |
| 101 |  | Dm-1 | Drosophila melanogaster | Insecta | 535 | NP_730039 | NM_168618 |  |
| 102 |  | Dm-2 |  |  | 809 | NP_729159 | NM_168162 |  |
| 103 |  | Dm-3 |  |  | 809 | AAQ22481* | BT010012* |  |
| 104 |  | Dm-4 |  |  | 721 | NP_652603 | NM_144346 |  |
| 105 |  | Am-1 | Apis mellifera | Insecta | 814 | XP_392428 | XM_392428 |  |
| 106 |  | Am-2 |  |  | 684 | XP_395231 | XM_395231 |  |
| 107 |  | Am-3 |  |  | 616 | XP_624170 | XM_624167 |  |
| 108 |  | Ae-1 | Aedes egypti | Insecta | 763 | EAT34965 | CH477932 |  |
| 109 |  | Ae-2 |  |  | 698 | EAT45610 | CH477264 |  |
| 110 |  | Tb-1 | Tribolium castaneum | Insecta | 610 | XP_970850 | XM_965757 |  |
| 111 |  | Tb-2 |  |  | 503 | XP_970907 | XM_965814 |  |
| 112 |  | Tb-3 |  |  | 487 | XP_970962 | XM_965869 |  |
| 113 |  | Tb-4 |  |  | 241 | XP_968277* | XM_963184* |  |
| 114 |  | Tb-5 |  |  | 649 | XP_968353* | XM_963260* |  |
| 115 |  | Dv-1 | Drosophila virilis | Insecta | 737 | ABD64823 | DQ378293 |  |
| 116 |  | Ce-1 | Caenorhabditis elegans | Secernentea | 612 | NP_493632 | NM_061231 |  |
| 117 |  | Ce-2 |  |  | 557 | NP_493631 | NM_061230 |  |
| 118 |  | Ce-3 |  |  | 540 | NP_502108 | NM_069707 |  |
| 119 |  | Ce-4 |  |  | 632 | NP_498717 | NM_066316 |  |
| 120 |  | Ce-5 |  |  | 405 | Q09379 | Q09379 |  |
| 121 |  | Ce-6 |  |  | 480 | NP_872102 | NM_182302 |  |
| 122 |  | Ce-7 |  |  | 1355 | T28715* | T28715* |  |
| 123 |  | Ce-8 |  |  | 499 | NP_502353 | NM_069952 |  |
| 124 |  | Ce-9 |  |  | 569 | NP_499895 | NM_067494 |  |
| 125 |  | Ce-10 |  |  | 395 | NP_502846 | NM_070445 |  |
| 126 |  | Ce-11 |  |  | 523 | NP_502524 | NM_070123 |  |
| 127 |  | Ce-12 |  |  | 453 | NP_499142* | NM_066741* |  |
| 128 |  | Ce-13 |  |  | 525 | NP_502007* | NM_069606* |  |
| 129 |  | Ce-14 |  |  | 405 | NP_505708 | NM_073307 |  |
| 130 |  | Ce-15 |  |  | 450 | NP_502523 | NM_070122 |  |
| 131 |  | Ce-16 |  |  | 413 | NP_501784 | NM_069383 |  |
| 132 |  | Ce-17 |  |  | 456 | NP_499263 | NM_066862 |  |
| 133 |  | Ce-18 |  |  | 613 | T16885 | T16885 |  |
| 134 |  | Ce-19 |  |  | 501 | NP_497218* | NM_064817* |  |
| 135 |  | Ce-20 |  |  | 512 | NP_493472* | NM_061071* |  |
| 136 |  | Ce-21 |  |  | 399 | NP_501000 | NM_068599 |  |
| 137 |  | Ce-22 |  |  | 387 | H89192 | H89192 |  |
| 138 |  | Ce-23 |  |  | 551 | NP_500411 | NM_068010 |  |
| 139 |  | Ce-24 |  |  | 884 | NP_500706* | NM_068305* |  |
| 140 |  | Ce-25 |  |  | 400 | NP_493480* | NM_061079* |  |
| 141 |  | Ce-26 |  |  | 444 | NP_493482 | NM_061081 |  |
| 142 |  | Ce-27 |  |  | 411 | NP_493478 | NM_061077 |  |
| 143 |  | Ce-28 |  |  | 806 | T15468 | T15468 |  |
| 144 |  | Ce-29 |  |  | 434 | NP_507036* | NM_074635* |  |
| 145 |  | Ce-30 |  |  | 602 | NP_493479 | NM_061078 |  |
| 146 |  | Ce-31 |  |  | 389 | NP_001023344* | NM_001028173* |  |
| 147 |  | Ce-32 |  |  | 530 | T28037 | T28037 |  |
| 148 |  | Cb-1 | Caenorhabditis briggsae | Secernentea | 612 | CAE62861 | CAAC01000031 |  |
| 149 |  | Cb-2 |  |  | 460 | CAE62025 | CAAC01000028 |  |
| 150 |  | Cb-3 |  |  | 629 | CAE66881 | CAAC01000060 |  |
| 151 |  | Cb-4 |  |  | 546 | CAE62860 | CAAC01000031 |  |
| 152 |  | Cb-5 |  |  | 566 | CAE56138* | CAAC01000178* |  |
| 153 |  | Cb-6 |  |  | 405 | CAE59819 | CAAC01000012 |  |
| 154 |  | Cb-7 |  |  | 499 | CAE59843 | CAAC01000013 |  |
| 155 |  | Cb-8 |  |  | 418 | CAE68089* | CAAC01000066* |  |
| 156 |  | Cb-9 |  |  | 455 | CAE65153* | CAAC01000045* |  |
| 157 |  | Cb-10 |  |  | 515 | CAE70840 | CAAC01000084 |  |
| 158 |  | Cb-11 |  |  | 461 | CAE64975 | CAAC01000045 |  |
| 159 |  | Cb-12 |  |  | 486 | CAE69222 | CAAC01000069 |  |
| 160 |  | Cb-13 |  |  | 450 | CAE58653 | CAAC01000009 |  |
| 161 |  | Cb-14 |  |  | 413 | CAE59969 | CAAC01000013 |  |
| 162 |  | Cb-15 |  |  | 400 | CAE64843 | CAAC01000044 |  |
| 163 |  | Cb-16 |  |  | 399 | CAE61514 | CAAC01000026 |  |
| 164 |  | Cb-17 |  |  | 434 | CAE61834 | CAAC01000028 |  |
| 165 |  | Cb-18 |  |  | 488 | CAE71802 | CAAC01000097 |  |
| 166 |  | Cb-19 |  |  | 341 | CAE63720* | CAAC01000034* |  |
| 167 |  | Cb-20 |  |  | 436 | CAE65680* | CAAC01000051* |  |
| 168 |  | Cb-21 |  |  | 533 | CAE67906* | CAAC01000064* |  |
| 169 |  | Cb-22 |  |  | 353 | CAE68207 | CAAC01000067 |  |
| 170 |  | Sp-1 | Strongylocentrotus purpuratus | Echinoidea | 644 | XP_786003 | XM_780910 |  |
| 171 |  | Sm-1 | Schistosoma mansoni | Trematoda | 392 | b* | b* |  |
| 172 |  | Sm-2 |  |  | 583 | b* | b* |  |
| 173 |  | Sm-3 |  |  | 504 | b* | b* |  |

^1^ Asteriks (*) indicate a partial or highly divergent sequence

^a^ Sequences were obtained from the: <http://genome.jgi-psf.org/ciona4/ciona4.home.html>

^b^ Sequences were obtained from the: <http://www.sanger.ac.uk/DataSearch/blast.shtml>

+ Sequences were used for calculation of functional divergence
