# Supplementary material for: Molecular evolution and functional divergence of the bestrophin protein family
Source: BMC Evol Biol. 2008 Feb 28;8:72. doi: 10.1186/1471-2148-8-72 (PMC2292144; doi:10.1186/1471-2148-8-72)
Supplement: Additional file 2 — Multiple sequence alignment of vertebrate bestrophins used for calculations of functional divergence. Protein sequences of 53 homologues of vertebrate bestrophins were aligned using the ClustalW algorithm. Shown is the amino acid sequence alignment after removal of gaps and highly divergent regions. Numbering is given according to human bestrophin 1. Sites with predicted functional divergence between bestrophin subfamilies are highlighted by vertical yellow boxes. [file 1471-2148-8-72-S2.pdf]

Multiple sequence alignment of vertebrate bestrophins used for the calculation of the functional divergence

```
                             10        20        30        40        50        60        70        80        90       100
                    ....|....|....|....|....|....|....|....|....|....|....|....|....|....|....|....|....|....|....|....|
HsB1                MTITYTSQVANARLGSFSRLLLCWRGSIYKLLYGEFLIFLLCYYIIRFIYRLALTEEQQLMFEKLTLYCDSYIQLIPISFVLGFYVTLVVTRWWNQYENL
PtB1                MTITYTSQVANARLGSFSRLLLCWRGSIYKLLYGEFLIFLLCYYIIRFIYRLALTEEQQLMFEKLTLYCDSYIQLIPISFVLGFYVTLVVTRWWNQYENL
MmB1                MTITYTSQVANARLGSFSRLLLCWRGSIYKLLYGEFFIFLLCYYIIRFIYRLALTEEQQLMFEKLTLYCDSYIQLIPISFVLGFYVTLVVTRWWNQYENL
MfB1                MTITYTSQVANARLGSFSRLLLCWRGSIYKLLYGEFFIFLLCYYIIRFIYRLALTEEQQLMFEKLTLYCDSYIQLIPISFVLGFYVTLVVTRWWNQYENL
BtB1                MTVTYSSQVANARLGSFSRLLLCWRGSIYKLLYGEFLIFLLCYYIIRFIYRMALTDEQQVIFEKLTLYCDSYIQLIPISFVLGFYVTLVVTRWWNQYENL
CfB1                MTVTYSSQVANARLGSFSRLLLCWRGSIYKLLYGEFLIFLLSYYTIRFIYRMALTEEQQVMFEKLTLYCDSYIQLIPISFVLGFYVTLVVTRWWNQYENL
MsB1                MTITYTNKVANARLGSFSSLLLCWRGSIYKLLYGEFLVFIFLYYSIRGLYRMVLSSDQQLLFEKLALYCDSYIQLIPISFVLGFYVTLVVSRWWSQYENL
RnB1                MTITYTNEVANARLGSFSCLLLRWRGSIYKLLYGEFLVFIFLYYCIRGLYRMVLSNHQQQQFEKLALYCNSYIQFIPISFVLGFYVTLVVSRWWNQYENL
MdB1                MTVTYTNRVANVHLGTFSRLLLQWRGSIYKLLYCEFCIFVLSYFTISIIYRLILSESQRLLFEKLALYCDKYAQLIPVSFVLGFYVTLVVSRWWSQYQSI
GgB1                MTVTYTNRVADARLGTFSQLLLQWKGSIYKLLYSEFLIFISLYFAISLVYRLILSESQRLMFEKLALYCNSYAELIPVSFVLGFYVSLVVSRWWAQYESI
Xt-1                MTVTYSNRVADARLGTFSRLLLRWRGSIYKLLYREFLIFISCYFIISVTYRFLLNEQQRIYFEKLALYCDNYAELIPVSFVLGFYVTLVVSRWWGQYESV
DrB1                MTVTYSRRVADARLGTFYRLLLRWKGSIYKLLYRELLIFTVMYCIISITYRCVLTEEQRRMFEKLSLYCDQYAQLIPVSFVLGFYVTLVVSRWWGQFESV
Tr-1                MTVTYSRRVADAGLGTFFHLLLRWKGSIYKLLYRELIIFSLLYSFFSIVYRFVLNNDQKRLFEKLAIYCDRYAELIPVSFVLGFYVTLVVSRWWGQFENV
Tn-1                MTVTYSRRVADAGLGTFFHLLLRWRGSIYKLLYRELLIFSLLYSFLSIVYRFVLNNEQRRLFEKLALYCDRYAELIPVSFVLGFYVTLVVSRWWGQFENV
Ol-1                MTVTYSRRVADAGLGTFFHLLLRWKGSIYKLLYRELIIFTLLYYFLSLVYRYMLDNDQRRLFERLSIYCDRYAELIPVSFVLGFYVTLVVSRWWGQFENI
HsB2                MTVTYTARVANARFGGFSQLLLLWRGSIYKLLWRELLCFLGFYMALSAAYRFVLTEGQKRYFEKLVIYCDQYASLIPVSFVLGFYVTLVVNRWWSQYLCM
PtB2                MTVTYTARVANARFGGFSQLLLLWRGSIYKLLWRELLCFLGFYMALSAAYRFVLTEGQKRYFEKLVIYCDQYASLIPVSFVLGFYVTLVVNRWWSQYLCM
MmB2                MTVTYTARVANARFGGFSQLLLLWRGSIYKLLWRELLCFLGFYMALSAAYRFVLTEGQKRYFEKLVIYCDQYASLIPVSFVLGFYVTLVVNRWWSQYLCM
BtB2                MTVTYTARVAKARFGGFSKLLLLWRGSIYKLLWRELLCFLGLFMALSAAYRFVLTEEQKRYFEKLVLYCDRYASLIPVSFVLGFYVTLVVHRWWNQYLSM
CfB2                MTVTYTARVANARFGGFSQLLLLWRGSIYKLLWRELVCFLGLYMALSAAYRFVLTEEQKRYFEKLVIYCDQYASLIPVSFVLGFYVTLVVHRWWNQYLCM
FcB2                MTVTYTARVANARFGGFSKLLLLWRGSIYKLLWRELLCFLGLYMALSAAYRFVLTEEQKRYFEKLVIYCDQYASLIPVSFVLGFYVTLVVHRWWNQYLCM
MsB2                MTVTYTARVANARFGGFSQLLLLWRGSIYKLLWRELLCFLGLYMALSAAYRFLLAEEQKRYFEKLVIYCDQYASLIPVSFVLGFYVTLVVHRWWNQYLCM
RnB2                MTVTYTARVANARFGGFSQLLLLWRGSIYKLLWRELLCFLGLYMALSAAYRFLLAEEQKRYFEKLVIYCDQYASLIPVSFVLGFYVTLVVHRWWNQYLCM
DrB2                MTVTYTARVANARFCGFSKLLLAWKGSIYKVLYKEFLAFFAMYTAISITYRFFLVDGQKRYFEKLSIYCDNYASLIPMSFVLGFYVTLVVNRWWSQYTSI
Tr-2                MTVTYTARVANARFCSFSKLLLAWKGSIYRLLYKEFLAFFALYAAISFTYRFFLDDDQKRYFEKLAIYCNHYSSLIPMSFVLGFYVTLVVNRWWSQYTSI
Ga-2                MTVTYTARVANARFCGFSKLLLAWRGSIYKVLYKEFLAFFAMYAAISITYRFLLFDDQKRYFEKLAIYCNHYASLIPMSFVLGFYVTLVVNRWWSQYTSI
Tn-2                MTVTYTARVANARFCSFSKLLLAWKGSIYRLLYKEFLAFFALYAAISFTYRFFLHNDQKRYFEKLAIYCNHYSSLIPMSFVLGFYVTLVVNRWWSQYTSI
Xt-2                MTVTYTARVANARFGGFYKLLLLWRGSIYKLLYKEFLAFFLMYLALSIIYRFFLNEEQKLYFDKVAIYCNNYANLIPVSFVLGFYVNLVVNRWWNQYLSL
HsB3                MTVTYSSKVANATFFGFHRLLLKWRGSIYKLLYREFIVFAVLYTAISLVYRLLLTGVQKRYFEKLSIYCDRYAEQIPVTFVLGFYVTLVVNRWWNQFVNL
PtB3                MTVTYSSKVANATFFGFHRLLLKWRGSIYKLLYREFIVFAVLYTAISLVYRLLLTGAQKRYFEKLSIYCDRYAEQIPVTFVLGFYVTLVVNRWWNQFVNL
MmB3                MTVTYSSKVANATFFGFHRLLFKWRGSIYKLLYREFIVFAVLYTAISLVYRLLLTGAQKRYFEKLSIYCDRYAEQIPVTFVLGFYVTLVVNRWWNQFVNL
LaB3                MTVTYSSKVANATFFGFHRLLLKWRGSIYKLLYREFIVFAILYTAISLVYRLLLTGAQKRYFEKLSIYCDRYAEQIPVTFVLGFYVTLVVNRWWNQFVNL
BtB3                MTVTYSSKVANATFFGFHRLLLKWRGSIYKLLYREFIVFAVLYTAISLMYRLLLTGAQKRFFEKLSIYCDRYAEQIPVTFVLGFYVTLVVNRWWNQFVNL
OaB3                MTVTYSSKVANATFFGFHRLLLKWRGSIYKLLYREFIAFAILYTAISLMYRLFLTGAQKRYFEKLSIYCDRYAEQIPVTFVLGFYVTLVVNRWWNQFVNL
MsB3                MTVTYSSKVANATFFGFHRLLLKWRGSIYKLLYREFIVFAVLYTAISLVYRLLLTGAQKRYFEKLSIYCDRYAEQIPVTFVLGFYVTLVVNRWWNQFVNL
RnB3                MTVTYSSKVANATFFGFHRLLLKWRGSIYKLLYREFIVFAVLYTAISLVYRLLLTGAQKRYFEKLSIYCDRYAEQIPVTFVLGFYVTLVVNRWWNQFVNL
MdB3                MTVTYSSKVANATFFGFHRLLLKWRGSIYKLLYREFIAFAILYTAISMMYRLLLTGAQKRYFEKLSIYCDRYAEQIPVTFVLGFYVTLVVNRWWNQFVNL
GgB3                MTVTYSSKVANATFFGFHRLLLKWKGSIYKLLYREFIVFATLYTAISVLYRFFLTGSQKRFFEKLSIYCDKYAEQIPVTFVLGFYVTLVVNRWWNQFVNL
Tr-3                MTVTYSSRVANASFFGFHRLLLRWRGSIYKLLYREFILFVVLYTIFSVIYRIVLLDHQKRLFEKLSLYFDKYAEQIPVTFVLGFYVTLVVNRWWNQFVNL
Xt-3                MTVTYSSKVANATFFGFHRLLLKWRGSIYKLLYREFLLFTSLYTTLSVIYRFFLTDSQKRYFEKVSMYCDKYAEQIPVTFVLGFYVTLVVNRWWNQFVNL
Ga-3                MTVTYSSKVANATFFSFHRLLLRWRGSIYKLLYREFVVFLALYTGLSVVYRLVLADHQKRLFEKLSMYCDKYAEQIPVTFVLGFYVTLVVNRWWHQFVNL
HsB4                MTVSYTLKVAEARFGGFSGLLLRWRGSIYKLLYKEFLLFGALYAVLSITYRLLLTQEQRYVYAQVARYCNRSADLIPLSFVLGFYVTLVVNRWWSQYTSI
PtB4                MTVSYTLKVAEARFGGFSGLLLRWRGSIYKLLYKEFLLFGALYAVLSITYRLLLTQEQRYVYAQVARYCNRSADLIPLSFVLGFYVTLVVNRWWSQYTSI
MmB4                MTVSYTLKVAEARFGGFSGLLLRWRGSIYKLLYKEFLLFGALYAVLSITYRLLLTQEQKHVYAQVARYCNRSADLIPLSFVLGFYVTLVVNRWWSQYTSI
BtB4                MTVSYTLKVAEARFGGFSGLLLRWRGSIYKLLYKEFLLFIALYALLSVTYRLLLTQEQKRVYAQVARYCNRSADLIPLSFVLGFYVTMVVNRWWAQYTSI
CfB4                MTVSYTLKVAEARFGGFSGLLLRWRGSIYKLLYKEFLLFTALYALLSITYRLLLTQEQRHVYAQVARYCNRSADLIPLSFVLGFYVTLVVNRWWAQYTSI
MlB4                MTVSYTLKVAEARFGGFSGLLLRWRGSIYKLLYKEFLLFTVLYALLSITYRLLLTQEQRLVYAQVARYCNRSADLIPLSFVLGFYVTLVVNRWWAQYTSI
OgB4                MTVSYTLKVAEARFGGFSGLLLRWRGSIYKLLYKEFLLFVALYALLSITYRLLLTQEQKHVYAQVARYCNRSADLIPLSFVLGFYVTLVVNRWWSQYTSI
RnB4                MTVSYTLKVAEARFGAFSGLLFRWRGSIYKLLYREFLLFCALYATLNITYRLLLTQEQKRIYAQVARYCNRSADLIPLSFVLGFYVTLVVNRWWSQYTSI
MdB4                MTVSYTLKVANARFGGFSALLFRWRGSIYKLLYKELLVFLGLYGVLSLTYRKLLSQEQKRIYAQVAQYCNRSADLIPMSFVLGFYVTLVVNRWWAQYTSI
GgB4                MTVSYTLKVANSRFGGFSKLLFRWKGSIYKLLYKEFIVFVVLYALLSIVYRRLLTEEQKRLYTKVAQYCNRSTDLIPMSFVLGFYVTLIVNRWWAQYTSI
Xt-4                MTVSYTLKVANASFGCFFKLLFRWKGSIYKLLYKEFIVFCCLYAMLSVTYRCLLNEKQKSEFAQLARYCNQYTDLIPVSFVLGFYVTLIVNRWWAQYTNI
Tr-4                MTVSYTQVVATVSFGGFSKLLFRWKGSIYKLLYKDLLVFCGIYLFFSLLYRFMLTPNQQDMFERIALYCDQFTNFIPIPFVLGFYVTAAFNRWWGQYTSF
Clustal Consensus   **::*:  **   :  *  **: *:****::*: ::  *   :  :   **  *   *:  : ::  * :   . **:.*******.  . *** *:  .
```
